# Analysis of Structural Determinants of Peptide MS 9a-1 Essential for Potentiating of TRPA1 Channel

**DOI:** 10.3390/md20070465

**Published:** 2022-07-21

**Authors:** Yulia A. Logashina, Kseniya I. Lubova, Ekaterina E. Maleeva, Viktor A. Palikov, Yulia A. Palikova, Igor A. Dyachenko, Yaroslav A. Andreev

**Affiliations:** 1 Shemyakin-Ovchinnikov Institute of Bioorganic Chemistry, Russian Academy of Sciences, ul. Miklukho-Maklaya 16/10, 117997 Moscow, Russia; yulia.logashina@gmail.com (Y.A.L.); lubova.ksenia@gmail.com (K.I.L.); katerina@1ns.ru (E.E.M.); 2 Institute of Molecular Medicine, Sechenov First Moscow State Medical University, Trubetskaya Str. 8, Bld. 2, 119991 Moscow, Russia; 3 Branch of the Shemyakin-Ovchinnikov Institute of Bioorganic Chemistry, Russian Academy of Sciences, Prospekt Nauki, 6, 142290 Pushchino, Russia; vpalikov@bibch.ru (V.A.P.); yuliyapalikova@bibch.ru (Y.A.P.); dyachenko@bibch.ru (I.A.D.)

**Keywords:** TRPA1, positive modulator, pain, inflammation, sea anemone, peptide, analgesic drugs

## Abstract

The TRPA1 channel is involved in a variety of physiological processes and its activation leads to pain perception and the development of inflammation. Peptide Ms 9a-1 from sea anemone *Metridium senile* is a positive modulator of TRPA1 and causes significant analgesic and anti-inflammatory effects by desensitization of TRPA1-expressing sensory neurons. For structural and functional analysis of Ms 9a-1, we produced four peptides—Ms 9a-1 without C-terminal domain (abbreviated as N-Ms), short C-terminal domain Ms 9a-1 alone (C-Ms), and two homologous peptides (Ms 9a-2 and Ms 9a-3). All tested peptides possessed a reduced potentiating effect on TRPA1 compared to Ms 9a-1 in vitro. None of the peptides reproduced analgesic and anti-inflammatory properties of Ms 9a-1 in vivo. Peptides N-Ms and C-Ms were able to reduce pain induced by AITC (selective TRPA1 agonist) but did not decrease AITC-induced paw edema development. Fragments of Ms 9a-1 did not effectively reverse CFA-induced thermal hyperalgesia and paw edema. Ms 9a-2 and Ms 9a-3 possessed significant effects and anti-inflammatory properties in some doses, but their unexpected efficacy and bell-shape dose–responses support the hypothesis of other targets involved in their effects in vivo. Therefore, activity comparison of Ms 9a-1 fragments and homologues peptides revealed structural determinants important for TRPA1 modulation, as well as analgesic and anti-inflammatory properties of Ms9a-1.

## 1. Introduction

Animal venoms have been used and studied by humankind since ancient times. Hunters and soldiers were armed with poison-tipped arrows, while ancient physicians used small amounts of animal venoms for wound healing and smallpox, leprosy, and fever treatment [1,2]. Venoms contain numerous biologically active compounds, including low-molecular-weight toxins, peptides, and enzymes. Such a natural mixture is a powerful tool, which enables animals to kill, while for science, it is a treasury of lifesaving medicines and analgesics. 

Long-term evolution has created a vast variety of peptide toxins, which selectively act on their physiological targets, such as ion channels and cell membrane receptors [3]. Currently, there are peptides from venoms that are already used as therapeutics (Ca_v_ channel blocker—chronic pain treatment) or are in clinical trials (ligands of K_v_1.3—autoimmune diseases’ therapy; Na_v_1.7 and nAChRs—pain treatment; TRPV6—tumors inhibition) [1]. However, the pool of potential therapeutic molecules is far from being exhausted.

Previously, we isolated and characterized a potent peptide modulator of transient receptor potential ankyrin 1 ion channel (TRPA1), τ-AnmTX Ms 9a-1, from the venom of sea anemone *Metridium senile* [4]. TRPA1 is a non-selective cationic channel expressed on the cell membrane of C-fiber neurons, epithelium cells of the respiratory tract, gastrointestinal tract, small blood vessels, and bladder, as well as in the skin, joints, and brain [5,6]. It is an important molecular target, engaged in many physiological processes, such as chemo-, mechano-, and thermosensitivity, and its activation leads to pain perception and the development of inflammatory conditions [5]. Besides, a gain-of-function TRPA1 mutation causes familial episodic pain syndrome that is characterized by episodes of exhausting upper body pain accompanied by starvation and physical exertion [7]. τ-AnmTX Ms 9a-1 (short name Ms 9a-1) modulates TRPA1 action in pathophysiological conditions and represents a new therapeutic strategy: this peptide potentiates TRPA1 causing desensitization of TRPA1-positive neurons and, as a result, suppresses neurogenic inflammation. Intravenous injection of Ms 9a-1 significantly reduced pain and inflammation in mice induced by TRPA1 agonist or Complete Freund’s Adjuvant (CFA) [4].

Peptide Ms 9a-1 consists of 35 amino acid residues with four cysteines that form two disulfide bonds, corresponding to the β-hairpin structure of class 9a of sea anemone peptides [8]. Primary structure analysis revealed poor homology to characterized sea anemone peptides [4]. Moreover, the gene coding target peptide contained two homologous molecules, named τ-AnmTX Ms 9a-2 (shortly Ms 9a-2) and τ-AnmTX Ms 9a-3 (Ms 9a-3). All peptides in precursor proteins (UniProt A0A1R3S3A8, SBO16029.1) were flanked by DP or EP processing sites for dipeptidyl peptidase proteolysis. Ms 9a-1 differs from Ms 9a-2 and Ms 9a-3 by the long C-terminal domain and amino acid residues in the middle region between the second and third cysteines [4]. Ms 9a-1 peptide could be the product of non-synonymous substitutions, domain recombination, or other gene mutations in the precursor genes [9]. Since the TRPA1 active fraction from the *M. senile* venom contained Ms 9a-1, we hypothesized that the differences between the peptides could be essential for the TRPA1 potentiation.

In this work, we analyzed the activity of Ms 9a-2 and Ms 9a-3 on TRPA1 and carried out Ms 9a-1 structural and functional analysis. For this purpose, we designed four peptides—Ms 9a-1 without C-terminal domain (abbreviated as N-Ms), short C-terminal domain Ms 9a-1 alone (C-Ms), as well as Ms 9a-2 and Ms 9a-3—and measured their activity in vitro and in vivo. We demonstrated that both parts of Ms 9a-1 and peptides Ms 9a-2 and Ms 9a-3 possessed a reduced potentiating effect on TRPA1. In animal models, fragments of Ms 9a-1 and homologous peptides demonstrated a selective mode of action; they reduced only pain induced by the TRPA1 agonist but not inflammatory response to Complete Freund’s Adjuvant (CFA). Homologous peptides Ms 9a-2 and Ms 9a-3 revealed analgesic and anti-inflammatory properties but possessed bell-shaped dose response and the absence of correlation with TRPA1 potentiation, which supports the hypothesis of other molecular targets’ existence for these peptides.

## 2. Results

### 2.1. Alignment of Peptides and Ms 9a-1 Spatial Structure Modeling

**Figure 1:**
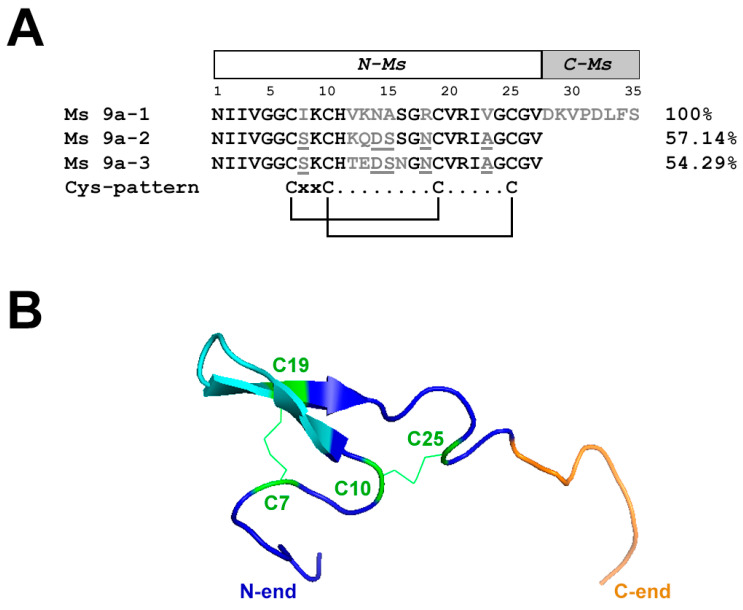
Alignment of Ms 9a homologues and spatial structure of Ms 9a-1. (**A**). Comparison of the amino acid sequences of Ms 9a-1, Ms 9a-2, and Ms 9a-3. Similar for all three peptides residues marked in black, variable regions marked in grey, mismatches in these regions underlined. Amino acids of Ms 9a-1 from 1 to 27 correspond to the engineered peptide N-Ms, while residues from 28 to 35 are C-Ms. (**B**). Spatial structure model of Ms 9a-1. Cys residues are green, the variable region between C10 and C19 is cyan, and non-homologous C-tail (С-Ms) is orange.

To identify potentially important residues of MS 9a-1 for TRPA1 channel binding, we aligned peptides Ms 9a-1, Ms 9a-2, and Ms 9a-3. In order to determine the mutual arrangement of Ms 9a-1 C-tail and the non-homologous region between the second and third Cys residues, we built a spatial structure of the peptide using AlphaFold2 Colab software considering NMR spectroscopy data for the homologous toxin Ugr 9a-1 (PDB code: 2LZO) [10]. Evidently, the most variable region in peptides Ms 9a-1-Ms 9a-3 is located in the central loop of the peptides (Figure 1A,B), whereas protruding C-terminus distinguishes Ms 9a-1 from Ms 9a-2 and Ms 9a-3 (Figure 1A,B and Supplementary Figure S1).

### 2.2. Peptide Production

Peptide Ms 9a-1 (35 a.a.) is distinguished from Ms 9a-2 and Ms 9a-3 (both 27 a.a.) by additional 8-amino acid residues in the C-terminal domain. Hence, we designed two peptides: Ms 9a-1 without C-terminal domain (N-Ms—MNIIVGGCIKCHVKNASGRCVRIVGCGV) and short C-terminal domain alone (C-Ms—DKVPDLFS). Moreover, Ms 9a-1 has a different amino acid sequence between the second and third cysteines, as well as a pair of point substitutions, compared to its homologues.

Short peptide C-Ms (8 a.a.) was a good candidate for the solid-phase peptide synthesis (SPPS), which provides high-efficiency production of peptides containing ~15 amino acids or less [11]. Synthesized C-Ms did not require any additional refolding procedures, as the peptide does not contain cysteine residues in its sequence.

To provide sufficient quantities of the other peptides, we used an *E. coli* expression system. The final yields of Ms 9a-1, N-Ms, Ms 9a-2, and Ms 9a-3 were estimated to be about 2.4, 1.3 mg, 4.0 mg, and 3.7 mg per liter of the *E. coli* culture, respectively.

### 2.3. Electrophysiology

**Figure 2:**
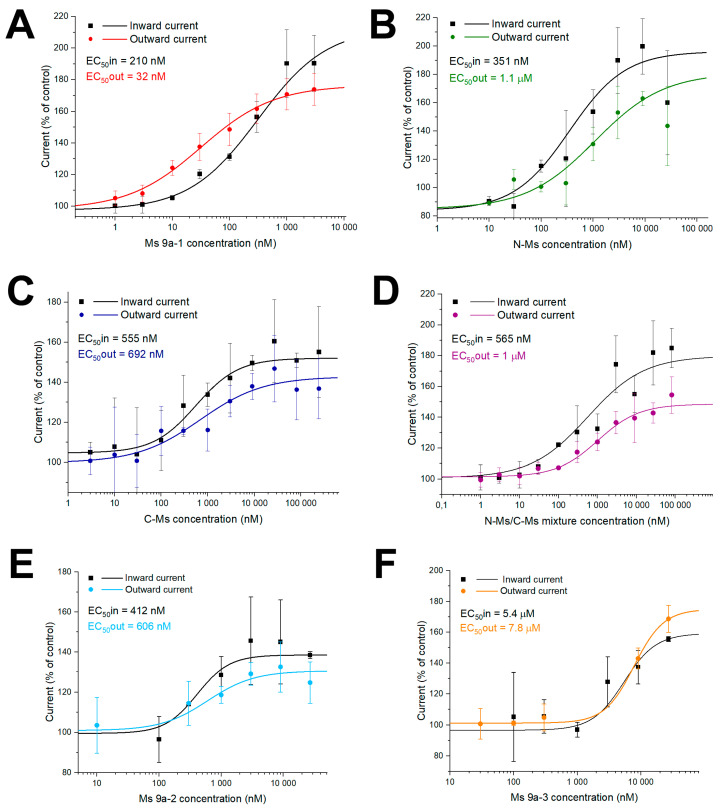
Action of Ms 9a-1 (**A**), N-MS (**B**), C-MS (**C**), N-MS/C-MS mixture (**D**), Ms 9a-2 (**E**), and Ms 9a-3 (**F**) on diclofenac-induced inward and outward TRPA1 currents. Each point is the mean ± SD of 4–7 measurements.

Peptide activity was measured in *Xenopus laevis* oocytes, heterologously expressing the TRPA1 channel (Figure 2 and Figure S2). Recordings occurred in repeated 4 s pulses of voltage ramp from −100 mV to 100 mV over 200 ms, whereby inward current was measured at −100 mV and outward at 100 mV. N-Ms and C-Ms also demonstrated different degrees of channel potentiation. The N-Ms peptide possessed similar effects to Ms 9a-1 on TRPA1 currents. They both potentiated diclofenac-induced inward currents up to 190%, with EC50 210 ± 3 nM and 351 ± 125 nM for Ms 9a-1 and N-Ms, respectively. Outward current potentiation induced by both peptides in the presence of diclofenac reached 175% and calculated EC50 of Ms 9a-1 and N-Ms were 32 ± 7 nM and 1.1 ± 0.8 µM, correspondingly (Figure 2A,B). C-terminal domain of Ms 9a-1, C-Ms, was less active, but also demonstrated potentiating effect on diclofenac-induced TRPA1 currents. C-Ms increased inward current up to 152%, while the outward current reached 143% of control, and EC50 was 555 ± 105 nM and 692 ± 230 nM for inward and outward currents, respectively (Figure 2C). 

We also measured the effect of a N-Ms and C-Ms mixture on TRPA1 activity to examine if it could reach the potentiation rate and effective concentrations of Ms 9a-1. However, the N-Ms+C-Ms mix possessed lower potentiating activity and constituted about 180% for inward and 145% for outward currents. The calculated EC50 of the mixture was 565 ± 186 nM and 1 ± 0.37 µM for inward and outward currents, correspondingly (Figure 2D). 

Both Ms 9a-2 and Ms 9a-3 were able to potentiate TRPA1 response to the agonist, but less efficiently and reliably than Ms 9a-1 (Figure 2E,F). Peptide Ms 9a-2 increased diclofenac-induced inward and outward current amplitudes up to 139% and 131%, respectively, and the calculated EC50 of inward current was 412 ± 48 nM, while for outward current, it was 606 ± 201 nM. Peptide Ms 9a-3 was more effective, potentiating inward and outward ion currents up to 159% and 175%, correspondingly, while half maximal effective concentrations of inward and outward currents were dramatically higher, taking 5.44 ± 2.9 µM and 7.8 ± 0.6 µM, correspondingly (Figure 2E,F).

### 2.4. Experiments In Vivo

Although peptide Ms 9a-1 significantly potentiates agonist-induced currents through TRPA1 in vitro, it does not cause pain or thermal hyperalgesia in vivo [4]. Ms 9a-1 (0.3 mg/kg) relieved pain, decreasing the duration of paw guarding by 78% and the number of paw licks by 55%, induced by TRPA1 activator AITC (allyl isothiocyanate), and it possesses an antinociceptive effect in the model of inflammation induced by Complete Freund’s Adjuvant test (CFA), reversing thermal hyperalgesia by 56% [4]. We compared the efficacy of peptides Ms 9a-1, C-Ms, N-Ms, Ms 9a-2, and Ms 9a-3 in models of AITC- and CFA-induced inflammatory pain. The dosage of peptides was chosen to provide molarities in blood similar to administration of 0.03, 0.1, and 0.3 mg/kg Ms 9a-1. 

**Figure 3:**
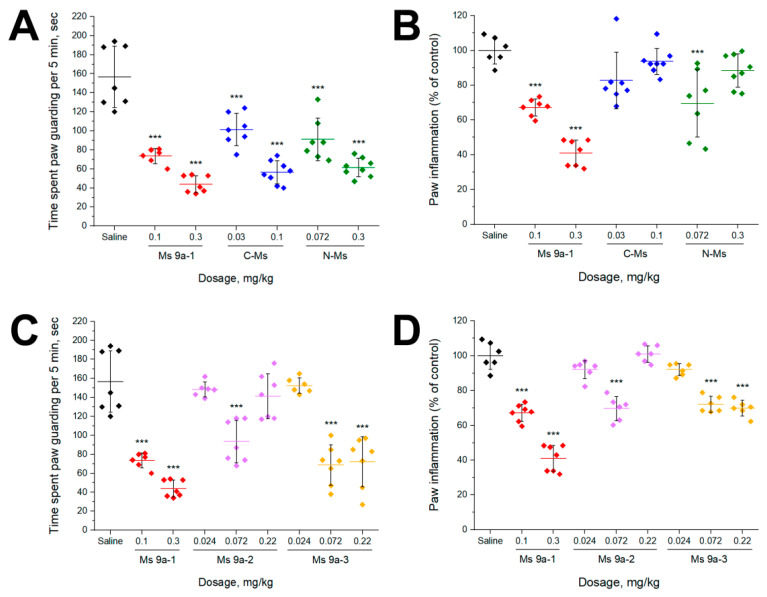
In vivo efficacy of peptides in AITC-induced pain model. (**A**,**C**) Effect of pretreatment with peptides on time spent paw guarding within a 5 min interval after AITC injection. (**B**,**D**) Effect of peptides on development of paw edema after AITC injection (4 h). The results are presented as the mean ± SD, n = 6–8 for each group; *** *p* < 0.001; versus saline group (ANOVA followed by a Tukey’s test).

AITC injection into the plantar surface of the hind paw induced licking and guarding of the paw, accompanied by swelling due to neurogenic inflammation. Intravenous (i.v.) administration of C-Ms and N-Ms significantly reduced nocifensive behavior after AITC injection similar to Ms 9a-1 decreasing the duration of paw guarding (~35–64%) (Figure 3A). Peptide Ms 9a-1 and N-MS (at 0.072 mg/kg) also significantly decreased the development of AITC-induced paw edema, while N-Ms at 0.3 mg/kg and C-Ms had no significant effect (Figure 3C). Ms 9a-2 revealed no significant effect at doses 0.024 and 0.22 mg/kg, but dosage 0.072 mg/kg decreased duration of paw guarding (~48%) and paw edema (~30%). Peptide Ms 9a-3 (at 0.072 and 0.22 mg/kg) was comparable to Ms 9a-1’s effects on AITC-induced nocifensive behavior, decreasing the time spent paw guarding (~55%) and reducing paw edema (~30%) (Figure 3D).

**Figure 4:**
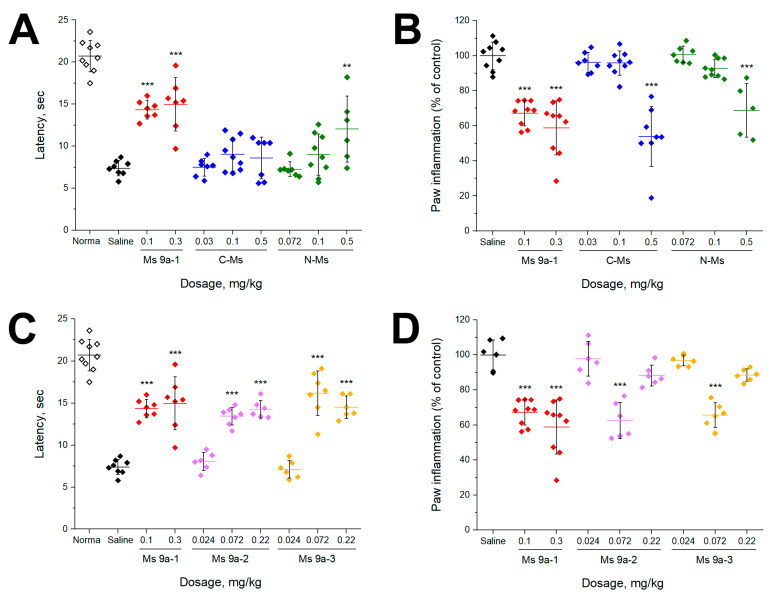
Efficacy of peptides in the CFA test. (**A**,**C**) The effect of peptides on paw withdrawal latency on a hot plate in the thermal hyperalgesia test after CFA injection. (**B**,**D**) Effect of peptides on CFA-induced paw edema. The results are presented as the mean ± SD; ***, *p* < 0.001; **, *p* < 0.01, versus saline group (ANOVA followed by a Tukey’s test), n = 5–9 for each group.

CFA test was used as a model of non-specific inflammation. CFA injection into the hind paw produced significant swelling and hyperalgesia within 24 h. Peptides were administrated 24 h after injection of CFA. Ms 9a-1 (0.1 and 0.3 mg/kg) significantly reversed thermal hyperalgesia, but C-Ms was not effective in the model and N-Ms was effective only at the highest dose tested (0.5 mg/kg) (Figure 4A). Surprisingly, both Ms 9a-2 and Ms 9a-3 reversed thermal hyperalgesia, similar to Ms 9a-1’s efficacy at corresponding doses (0.072 and 0.22 mg/kg) (Figure 4C). We also evaluated the ability of peptides to reduce development of paw edema 24 h after administration. Ms 9a-1 (0.1 and 0.3 mg/kg) significantly (~35–39%) decreased paw edema, but its fragments (C-Ms and N-Ms) were able to produce a similar effect only at doses 0.5 mg/kg. Ms 9a-2 and Ms 9a-3 significantly reduced the development of paw edema by 37% and 34%, respectively, but only at dose 0.072 mg/kg (Figure 4D).

## 3. Discussion

TRPA1 is a non-selective cationic channel, the dysfunction of which is associated with many diseases, such as cough, asthma, ulcerative colitis, pancreatitis, arthritis, diabetic neuropathy, ischemia, multiple sclerosis, stroke, and others [5,6]. It initiates pain signals triggered by mechanical exposure, temperature changes, and chemical irritants. TRPA1 is an extremely promising target for novel drug development, as it is almost the only channel in the sensory system that perceives nearly all types of harmful stimuli. For example, TRPA1 antagonists decrease the response of sensory neurons to noxious mechanical stimulation in inflammatory conditions, trauma, or osteoarthritis [12]. At the same time, TRPA1-deficient mice showed controversial effects on intensive mechanical stimuli [13,14]. Therefore, the channel is not a key mechanotransducer, but it is undoubtedly involved in the development of mechanical allodynia and hypersensitivity. The same holds for thermosensitivity: TRPA1 is involved in cold allodynia, but it is not a major cold sensor like TRPM8 [15,16]. TRPA1 is also activated by various exogenous compounds, such as allyl isothiocyanate (AITC) [17] (the pungent component of mustard, radish, horseradish, and wasabi), other substances of plant origin [18], acrolein [17] (from exhaust gases), chloroacetophenone [19] (the irritant component of tear gas), lidocaine [20], diclofenac [21], as well as by endogenous ligands accompanying inflammation and chronic pain [22,23,24]. 

TRPA1 modulators have been used in traditional medicine as anti-inflammatory drugs since ancient times, acting on TRPA1 in different ways. One analgesic strategy is the use of TRPA1 agonists. Mustard plaster containing AITC, when applied topically, triggers a burning sensation by channel activation, and the following TRPA1 desensitization decreases the infiltration of pro-inflammatory agents and inflammation. Besides, the analgesic effect of a widely used anti-inflammatory drug, acetaminophen, is also driven by TRPA1 activation [25]. In 2018, Flex Pharma published the first results of an exploratory Phase 2 study for multiple sclerosis of FLX-787, a TRPA1/TRPV1 co-activator [26]. They reported FLX-787 was well tolerated, safe, and effective in treating muscle spasm frequency, pain, and spasticity. The more appropriate strategy to abolish pain seems to be the usage of TRPA1 antagonists. However, no selective natural inhibitors of TRPA1 have been found so far and synthetic compounds have multiple side effects and cannot be used as medicines [27,28,29,30]. The search for such compounds is still underway: Glenmark Pharmaceuticals Ltd in 2016 reported about the beginning of Phase 2 clinical trials for neuropathic pain and respiratory disorders of GRC 17536, a synthetic TRPA1 channel antagonist. The compound was effective in asthma models, attenuated chronic obstructive pulmonary disease, and reduced peripheral diabetic neuropathic pain [31].

The last class of TRPA1 ligands are positive modulators that increase the channel response to agonist application. Currently, two peptides with such activity, Ms 9a-1 [4] and Ueq 12-1 (τ-AnmTx Ueq 12-1 from *Urticina eques*) [32], are known. They were isolated from sea anemone venoms and possess potentiating activity on TRPA1 with a similar to weak agonist mechanism of analgesic and anti-inflammatory action: channel potentiation causes desensitization of TRPA1-positive neurons and, as a result, reduces voltage-gated calcium and sodium currents in sensory neurons, suppressing neurogenic inflammation. Both peptides reduced nocifensive behavior and paw edema induced by TRPA1 agonist AITC, as well as decreasing non-specific inflammation caused by the injection of CFA. Peptide Ueq 12-1 forms a defensin-like fold and, in addition to potentiating activity against TRPA1, also exhibits an antibacterial effect. Cnidarians contain a variety of small, cysteine-rich peptides with a range of biological activities, including antimicrobial peptides, neurotoxins, and enzyme inhibitors, all of which are distantly related to vertebrate defensins [33]. Interestingly, the Ms 9a-1 peptide has a different structure and belongs to the class 9a of sea anemone peptides, with four Cys residues and a boundless β-hairpin fold. The protein organization of class 9a toxin precursors is similar: a signal peptide and a propeptide are followed by several homologous toxins separated by proteolytic sites [4,10]. Ms 9a-1 revealed only structural homology to the known sea anemone peptides. In addition, two homologous peptides, Ms 9a-2 and Ms 9a-3, were predicted in protein precursors of Ms 9a-1, which share 54–57% of identity (Figure 1A). Ms 9a-1 distinguishes from Ms 9a-2 and Ms 9a-3 by the long C-terminal domain and the region between the second and third cysteine residues in the sequence. We assumed that these non-homologous residues could be responsible for the peptide activity on the TRPA1 channel. We also decided to examine whether the C-terminal domain is essential for peptide activity or it possesses its own activity on the TRPA1 channel. We also evaluated the effects of Ms 9a-2 and Ms 9a-3 on TRPA1 activity to confirm the importance of the long C-terminal tail for the potentiating effect of Ms 9a-1. The electrophysiological study on *X. laevis* oocytes expressing TRPA1 revealed lower and instable potentiating effects of Ms 9a-2 and Ms 9a-3 compared to Ms 9a-1 on the TRPA1 channel. The maximal effect of Ms 9a-2 was 45–50% lower than that of Ms 9a-1 and effective concentrations increased 3- and 10-times for inward and outward currents, respectively. Peptide Ms 9a-3 demonstrated a 15–30% reduction in potentiation compared to Ms 9a-1, the EC50 for inward current was much greater (17 times), and effective concentration for potentiation of outward currents was more than two orders of magnitude higher. Both N-Ms and C-Ms were still able to potentiate diclofenac-induced currents mediated by TRPA1. They had higher effective concentrations for inward current than Ms 9a-1, but of the same order of magnitude. At the same time, for TRPA1 outward currents, the effective concentrations of both fragments increased by more than an order of magnitude. These data indicate the importance of the C-terminal tail for peptide potency on outward currents through TRPA1, as well as suggesting that amino acids between the second and third Cys residues form the region for binding to the channel surface, and the substitutions in this sequence lead to the changes in inward current potentiating activity, as in the case of Ms 9a-2 and Ms 9a-3.

The modeling of Ms 9a-1 spatial structure based on NMR spectroscopy data for the homologous toxin Ugr 9a-1 [10] revealed that the C-tail and the middle non-homologous region between second and third cysteines could form two “hands”, most probably binding separate regions of the TRPA1 extracellular surface. Interaction of these “hands” with the open channel shifts the kinetic equilibrium and increases the magnitude of the currents mediated by TRPA1. Interestingly, even one “hand” was able to potentiate channel activity, meaning Ms 9a-1 has two binding sites on the TRPA1 surface for positive modulation. According to electrophysiological data, effective potentiation required the binding of both “hands”, so we assumed that the mixture of N-Ms and C-Ms could mimic the Ms 9a-1 activity. However, the mixture of N-Ms+C-Ms was less effective than the native peptide. Therefore, the mutual arrangement of the Ms 9a-1 C-tail and the non-homologous region between the second and third Cys residues is responsible for the proper binding to the TRPA1 surface, mediating a significant potentiating action. Both the N-terminal and C-terminal domains are essential for Ms 9a-1 activity. We assumed that the mixture could be less active than natural peptide due to the incorrect spatial arrangement of N-Ms and C-Ms amino acid residues and/or the inconstant structure of polypeptide composition.

According to our previous study [4], Ms 9a-1 possesses unique properties; it acts as a positive modulator in vitro, significantly increasing TRPA1 response to agonists, and produces powerful analgesic effects in vivo. Ms 9a-1 significantly reduced AITC-induced nociceptive behavior and the development of inflammation. Peptides C-Ms, N-Ms, and Ms 9a-3, also demonstrated antinociceptive effects (Figure 3), decreasing the duration of paw guarding to the same extent as Ms 9a-1 (Figure 2). Peptide Ms 9a-2 reduced nociceptive behavior only at dose 0.072 mg/kg and did not reveal any significant analgesic effect at 0.024 and 0.22 mg/kg doses, showing so-called “bell-shaped” or “inverse U-shaped” dose response (Figure 3C), which correlates with electrophysiology data, where Ms 9a-2 demonstrated the lowest potentiating activity. Despite efficacy of peptides in alleviation of AITC pain effects, almost all of them had a weak effect on paw edema development. Only Ms 9a-3 was capable of significantly and steadily reducing AITC-induced development of paw edema at the same level as Ms 9a-1. Thus, peptides C-Ms, N-Ms, and Ms 9a-2 are able to block pain transmission but failed to prevent vasoactive peptide (Substance P and CGRP) release from nociceptors. Therefore, peptides C-Ms, N-Ms, and Ms 9a-2 are unable to desensitize TRPA1-expressing neurons to provide robust analgesic and anti-inflammatory effects.

Additionally, peptides C-Ms and N-Ms did not reverse CFA-induced thermal hyperalgesia, and only N-Ms (0.5 mg/kg) had scattered but statistically significant effect (Figure 4A). C-Ms and N-Ms at doses 0.5 mg /kg were able to reduce paw edema compared to control, while lower doses were ineffective (Figure 4B). Thus, neither C-Ms nor N-Ms could reproduce analgesic and anti-inflammatory properties of Ms 9a-1. 

Ms 9a-2 and Ms 9a-3 significantly reversed CFA-induced thermal hyperalgesia with equal to Ms 9a-1 potency that does not correspond to their weak potentiating effect in vitro (Figure 4C). They decreased CFA-induced paw edema only at one dose (0.072 mg/kg), showing a “bell-shaped” or “inverse U-shaped” dose response curve (Figure 4D). The most probable mechanism of this phenomenon in this case is the peptides’ action on different receptors/channels, depending on the concentration and the following competition between multiple signal pathways [34,35,36]. Taken together, data on peptides Ms 9a-2 and Ms 9a-3 show that these peptides can interact with TRPA1, but it is not their key target in vivo. Most probably, these peptides affect several molecular targets that can lead to significant analgesic and anti-inflammatory effects at some doses. Evidently, these peptides are less effective in potentiating TRPA1 but can reduce nocifensive behavior in vivo with equal potency with Ms 9a-1.

## 4. Materials and Methods

### 4.1. Synthesis of C-Ms

The short peptide C-Ms (8 a.a.—DKVPDLFS) was synthesized similarly to Osmakov et al. (2019) by the solid-phase method, using Fmoc-protected amino acids (Iris Biotech GmbH, Marktredwitz, Germany) and O-HATU as a condensing reagent. The peptide was deprotected and cleaved from the Tentagel HL-NH 2 resin by treatment with TFA/DTT/H_2_O/di-n-butylmagnesium (150/4/3/0.5 *w/w*) cocktail. The peptide purification was performed on Triart-C18 column (120 Å, 10 μm, 150 × 30 mm, YMC, Kyoto, Japan), using HPLC system (333/334 pump with a 215 liquid handler, Gilson, UK) with spectrophotometric detection at 210 and 280 nm. The peptide was separated in a linear gradient of an acetonitrile/water solution and about 2 mg of pure C-Ms was obtained. The purity of C-Ms was confirmed by ESI-MS analysis. All reagents and solvents used without additional purification were purchased from Acros Organics (Thermo Fisher Scientific, New Jersey, NJ, USA) and Sigma-Aldrich (Merck KGaA, Darmstadt, Germany).

### 4.2. Recombinant Peptide Production

Recombinant peptides were produced linked to a thioredoxin domain using an *E. coli* BL21(DE3) express strain. Competent cells were transformed with the expression vector and then cultivated at 37 °C in LB medium containing ampicillin at a concentration of 100 μg/mL. Expression was induced when the culture density reached A600 ~ 0.6–0.8 by adding isopropyl-1-thio-β-D-galactopyranoside (IPTG) up to 0.2 mM. The cells were cultivated for 18 h at 25 °C, then harvested by centrifuging (5 min at 6000× *g*), re-suspended in a buffer for metal affinity chromatography (400 mM NaCl, 20 mM Tris-HCl, pH 7.5), ultrasonicated and centrifuged (15 min at 9000× *g*) to remove all insoluble particles. The supernatant was applied to a HisPur™ Ni-NTA metal affinity resin (Thermo Scientific, Waltham, MA, USA) preliminarily equilibrated with start buffer (400 mM NaCl, 20 mM Tris-HCl, pH 7.5), washed with the buffer containing 25 mM imidazole, 400 mM NaCl, 20 mM Tris–HCl, pH 7.5, and eluted with the elution buffer (250 mM imidazole, 400 mM NaCl, 20 mM Tris–HCl, pH 7.5). Thioredoxin fusion proteins were diluted to 1 mg/mL and cleaved overnight in the dark at room temperature by CNBr with the addition of HCl up to 0.2 M, as described previously [37]. The molar ratio of CNBr to fusion proteins was adjusted to 600:1. Recombinant peptides were isolated from the reaction mixture by a reverse-phase HPLC on Jupiter C5 column (300 Å, 10 μm, 250 × 10 mm, Phenomenex, Torrance, CA, USA) in a linear gradient of MeCN/0.1% TFA solution from 0% to 60% over 60 min and constant flow rate 5 mL/min. The purity of target peptides was confirmed by MALDI-TOF mass spectrometry and N-terminal sequencing.

### 4.3. Electrophysiological Studies on Xenopus Laevis Oocytes

Oocytes expressing rat TRPA1 (Q6RI86) channel were prepared as described previously [4]. Experiments were approved by the Institutional Animal Care and Use Committee (IACUC) of the Shemyakin–Ovchinnikov Institute of Bioorganic Chemistry Russian Academy of Sciences (Protocol Number 267/2018; date of approval: 28 February 2019). Female frogs were anaesthetized with 0.17% solution of tricaine methane sulfonate (MS222) and oocytes were surgically removed. cRNA was synthesized using a HiScribe™ T7 High Yield RNA Synthesis Kit (New England Biolabs, Ipswich, MA, USA) according to the manufacturers’ protocol for capped transcripts and then injected in *X. laevis* oocytes. The oocytes were kept for 2–7 days at 16–17 °C in sterile ND-96 medium (96 mM NaCl, 2 mM KCl, 1.8 mM CaCl2, 1 mM MgCl_2_, 5 mM HEPES, pH 7.4) supplemented with 50 μg/mL gentamycin. Electrophysiological recordings were carried out at room temperature (22–24 °C) in Ca^2+^-free solution (96 mM NaCl, 2 mM KCl, 1 mM MgCl_2_, 5 mM HEPES, pH 7.4) using the GeneClamp500 amplifier (Axon Instruments, Union City, CA, USA) at a holding potential of −50 mV. The data were filtered at 20 Hz and digitized at 100 Hz by an AD converter L780 (LCard, Moscow, Russia) using in-house software. Oocytes were impaled with two glass microelectrodes filled with 3 M KCl. Inward/outward currents were recorded at repeated every 4 seconds a stimuli comprising step from holding potential to −100 mV for 80 ms followed by voltage ramp from −100 mV to +100 mV for 200 ms and final step to holding potential. Diclofenac (300 μM in Ca^2+^-free solution) [21] was used to activate the channel. Diclofenac has no effect on uninjected oocytes of *X. laevis* but causes current increases in TRPA1-expressing oocytes with EC_50_ 210 μM [21].

### 4.4. Computation and Structure Modeling

The similarity of Ms 9a-1 sequence to peptides Ms 9a-2 and Ms 9a-3, as well as class 9a of sea anemone peptides, was determined in previous work [4]. The proposed spatial structure of Ms 9a-1 was built by AlphaFold2 Colab (ColabFold: AlphaFold2 using MMseqs2, V 1.4, Korean Bioinformation Center, Seoul, South Korea) at default settings [38] and structural elements were illustrated by using PyMOL Molecular Graphics System (Version 2.3.2 Schrödinger^®^, LLC, New York, NY, USA).

### 4.5. In Vivo Experiments

#### 4.5.1. Animal Models

The study fully conforms to the World Health Organization’s International Guiding Principles for Biomedical Research Involving Animals. All experiments were approved by the Institutional Commission for the Control and Use of Laboratory Animals of the Branch of the Shemyakin–Ovchinnikov Institute of Bioorganic Chemistry of the Russian Academy of Sciences (protocol number: IACUC No. 891/22, date of approval: 17 May 2022). Adult male CD-1 mice (weight ~30 g) were housed at room temperature (23 ± 2 °C) and subjected to a 12 h light–dark cycle with food and water available ad libitum. Peptides were dissolved in saline. The significance of the data was determined by analysis of variance (ANOVA) followed by Turkey’s post hoc test. Data are presented as mean ± SD. 

#### 4.5.2. Allyl Isothiocyanate (AITC)-Induced Nocifensive Beaviour

To produce nocifensive behavioral response, TRPA1 agonist AITC (20 μL, 0.5% in saline) was injected into the plantar surface of the hind paw. Peptides or saline were administrated i.v. 30 min before AITC administration. The diameter of the paw was evaluated using an electronic digital caliper before the test and 2, 4, and 24 h after injection. The duration of paw guarding was recorded for 5 min after injection of AITC. Percent inflammation was calculated according to the formula ((post-dose paw diameter − before AITC paw diameter)/(control post-dose paw diameter − control before AITC paw diameter)) × 100%.

#### 4.5.3. Complete Freund’s Adjuvant (CFA)-Induced Inflammation and Thermal Hyperalgesia

Test was induced using CFA suspended in an oil/saline (1:1) emulsion. To induce paw inflammation and thermal hyperalgesia, mice were injected with 20 μL of CFA emulsion into the plantar surface of the left hind paw. The control mice were injected with 20 μL of saline. After 24 h, peptides or saline were i.v. administrated. Paw withdrawal latency was recorded using a hot plate (53 °C) 30 min after peptide or saline administration. The diameter of the paw was evaluated before CFA injection, before peptides or saline administration and 2, 4, and 24 h after peptides or saline administration using electronic digital caliper. Percent inflammation was calculated according to the formula ((post-dose paw diameter − before CFA paw diameter)/(control post-dose paw diameter − control before CFA paw diameter)) × 100%.

## 5. Conclusions

Ms 9a-1 is the positive modulator of TRPA1 and can attenuate pain responses induced by TRPA1 agonists and inflammation. In this work, we demonstrated that fragments of Ms 9a-1—N-Ms and C-Ms—were able to potentiate channel activity in vitro. Therefore, both the N-terminal and C-terminal domains participate in Ms 9a-1 binding to the channel and contribute to the modulation of its activity. Additionally, N-Ms and homologous peptides Ms 9a-2 and Ms 9a-3 possessed different magnitudes of TRPA1 modulation. Therefore, the region between the second and third Cys residues and the C-tail of Ms 9a-1 are responsible for proper TRPA1 channel binding. The N-terminal part (N-Ms) and the C-tail (C-Ms) of Ms 9a-1 possessed no or weak analgesic and anti-inflammatory properties in a CFA-induced model of inflammation, highlighting the importance of their cooperating binding for potent effects in vivo. Peptides Ms 9a-2 and Ms 9a-3 showed low TRPA1 potentiating potency, but also revealed analgesic properties. Further studies are needed to reveal the molecular targets of these peptides and mechanisms for pain attenuation.

## Data Availability

Not applicable.

## Supplementary Materials

The following supporting information can be downloaded at: https://www.mdpi.com/article/10.3390/md20070465/s1, Figure S1: AlphaFold2 Colab quality plots for spatial structure model of Ms9a-1., Figure S2: Action of peptides on diclofenac-induced inward and outward TRPA1 currents. Representative traces.

## Abbreviations

AITC	allyl isothiocyanate
CFA	Complete Freund’s adjuvant
C-Ms	short C-terminal domain of Ms 9a-1
Ms 9a-1 (full name—τ-AnmTX Ms 9a-1)	*Metridium senile* peptide of structural class 9a of sea anemone peptides, member 1
Ms 9a-2 (full name—τ-AnmTX Ms 9a-2)	*Metridium senile* peptide of structural class 9a of sea anemone peptides, member 2
Ms 9a-3 (full name—τ-AnmTX Ms 9a-3)	*Metridium senile* peptide of structural class 9a of sea anemone peptides, member 3
N-Ms	Ms 9a-1 without C-terminal domain
nAChR	Neuronal nicotinic acetylcholine receptor
TRPV6	Transient Receptor Potential channel, subfamily V (vanilloid), member 6
TRPM8	Transient Receptor Potential channel, subfamily M (melastatin), member 8
TRPA1	transient receptor potential cation channel, subfamily A (ankyrin), member 1
Ugr 9a-1 (full name—π-AnmTX Ugr 9a-1)	*Urticina grebelnyi* peptide of structural class 9a of sea anemone peptides, member 1

## References

[1] Pennington M.W., Czerwinski A., Norton R.S. (2018). Peptide Therapeutics from Venom: Current Status and Potential. Bioorg. Med. Chem..

[2] Utkin Y.N. (2015). Animal Venom Studies: Current Benefits and Future Developments. World J. Biol. Chem..

[3] Zhang Y. (2015). Why Do We Study Animal Toxins?. Zool. Res..

[4] Logashina Y.A., Mosharova I.V., Korolkova Y.V., Shelukhina I.V., Dyachenko I.A., Palikov V.A., Palikova Y.A., Murashev A.N., Kozlov S.A., Stensvåg K. (2017). Peptide from Sea Anemone Metridium Senile Affects Transient Receptor Potential Ankyrin-Repeat 1 (TRPA1) Function and Produces Analgesic Effect. J. Biol. Chem..

[5] Logashina Y.A., Korolkova Y.V., Kozlov S.A., Andreev Y.A. (2019). TRPA1 Channel as a Regulator of Neurogenic Inflammation and Pain: Structure, Function, Role in Pathophysiology, and Therapeutic Potential of Ligands. Biochemistry.

[6] Samanta A., Hughes T.E.T., Moiseenkova-Bell V.Y. (2018). Transient Receptor Potential (TRP) Channels. Membrane Protein Complexes: Structure and Function.

[7] Kremeyer B., Lopera F., Cox J.J., Momin A., Rugiero F., Marsh S., Woods C.G., Jones N.G., Paterson K.J., Fricker F.R. (2010). A Gain-of-Function Mutation in TRPA1 Causes Familial Episodic Pain Syndrome. Neuron.

[8] Kozlov S., Grishin E. (2012). Convenient Nomenclature of Cysteine-Rich Polypeptide Toxins from Sea Anemones. Peptides.

[9] Kini R.M. (2018). Accelerated Evolution of Toxin Genes: Exonization and Intronization in Snake Venom Disintegrin/Metalloprotease Genes. Toxicon.

[10] Osmakov D.I., Kozlov S.A., Andreev Y.A., Koshelev S.G., Sanamyan N.P., Sanamyan K.E., Dyachenko I.A., Bondarenko D.A., Murashev A.N., Mineev K.S. (2013). Sea Anemone Peptide with Uncommon β-Hairpin Structure Inhibits Acid-Sensing Ion Channel 3 (ASIC3) and Reveals Analgesic Activity. J. Biol. Chem..

[11] Bray B.L. (2003). Large-Scale Manufacture of Peptide Therapeutics by Chemical Synthesis. Nat. Rev. Drug Discov..

[12] McGaraughty S., Chu K.L., Perner R.J., Didomenico S., Kort M.E., Kym P.R. (2010). TRPA1 Modulation of Spontaneous and Mechanically Evoked Firing of Spinal Neurons in Uninjured, Osteoarthritic, and Inflamed Rats. Mol. Pain.

[13] Kwan K.Y., Glazer J.M., Corey D.P., Rice F.L., Stucky C.L. (2009). TRPA1 Modulates Mechanotransduction in Cutaneous Sensory Neurons. J. Neurosci..

[14] Petrus M., Peier A.M., Bandell M., Hwang S.W., Huynh T., Olney N., Jegla T., Patapoutian A. (2007). A Role of TRPA1 in Mechanical Hyperalgesia Is Revealed by Pharmacological Inhibition. Mol. Pain.

[15] Knowlton W.M., Bifolck-Fisher A., Bautista D.M., McKemy D.D. (2010). TRPM8, but Not TRPA1, Is Required for Neural and Behavioral Responses to Acute Noxious Cold Temperatures and Cold-Mimetics In Vivo. Pain.

[16] Moparthi L., Survery S., Kreir M., Simonsen C., Kjellbom P., Hogestatt E.D., Johanson U., Zygmunt P.M. (2014). Human TRPA1 Is Intrinsically Cold- and Chemosensitive with and without Its N-Terminal Ankyrin Repeat Domain. Proc. Natl. Acad. Sci. USA.

[17] Hinman A., Chuang H.H., Bautista D.M., Julius D. (2006). TRP Channel Activation by Reversible Covalent Modification. Proc. Natl. Acad. Sci. USA.

[18] Nassini R., Materazzi S., Vriens J., Prenen J., Benemei S., De Siena G., la Marca G., Andre E., Preti D., Avonto C. (2012). The “headache Tree” via Umbellulone and TRPA1 Activates the Trigeminovascular System. Brain.

[19] Bessac B.F., Sivula M., von Hehn C.A., Caceres A.I., Escalera J., Jordt S.E. (2009). Transient Receptor Potential Ankyrin 1 Antagonists Block the Noxious Effects of Toxic Industrial Isocyanates and Tear Gases. FASEB J..

[20] Leffler A., Lattrell A., Kronewald S., Niedermirtl F., Nau C. (2011). Activation of TRPA1 by Membrane Permeable Local Anesthetics. Mol. Pain.

[21] Hu H., Tian J., Zhu Y., Wang C., Xiao R., Herz J.M., Wood J.D., Zhu M.X. (2010). Activation of TRPA1 Channels by Fenamate Nonsteroidal Anti-Inflammatory Drugs. Pflug. Arch..

[22] Trevisan G., Hoffmeister C., Rossato M.F., Oliveira S.M., Silva M.A., Silva C.R., Fusi C., Tonello R., Minocci D., Guerra G.P. (2014). TRPA1 Receptor Stimulation by Hydrogen Peroxide Is Critical to Trigger Hyperalgesia and Inflammation in a Model of Acute Gout. Free Radic. Biol. Med..

[23] Andersson D.A., Gentry C., Moss S., Bevan S. (2008). Transient Receptor Potential A1 Is a Sensory Receptor for Multiple Products of Oxidative Stress. J. Neurosci..

[24] Motter A.L., Ahern G.P. (2012). TRPA1 Is a Polyunsaturated Fatty Acid Sensor in Mammals. PLoS ONE.

[25] Andersson D.A., Gentry C., Alenmyr L., Killander D., Lewis S.E., Andersson A., Bucher B., Galzi J.L., Sterner O., Bevan S. (2011). TRPA1 Mediates Spinal Antinociception Induced by Acetaminophen and the Cannabinoid Δ^9^-Tetrahydrocannabiorcol. Nat. Commun..

[26] FLX-787 Significantly Reduces Muscle Cramp/Spasm Frequency and Improves Spasticity in a Phase 2 Study (Flex-201) in Patients with Multiple Sclerosis. ECTRIMS Online Library. Short G. 10 October 2018; 228473. https://onlinelibrary.ectrims-congress.eu/ectrims/2018/ectrims-2018/228473/glenn.short.flx-787.significantly.reduces.muscle.cramp.spasm.frequency.and.html.

[27] Eid S.R., Crown E.D., Moore E.L., Liang H.A., Choong K.C., Dima S., Henze D.A., Kane S.A., Urban M.O. (2008). HC-030031, a TRPA1 Selective Antagonist, Attenuates Inflammatory- and Neuropathy-Induced Mechanical Hypersensitivity. Mol. Pain.

[28] Wei H., Hamalainen M.M., Saarnilehto M., Koivisto A., Pertovaara A. (2009). Attenuation of Mechanical Hypersensitivity by an Antagonist of the TRPA1 Ion Channel in Diabetic Animals. Anesthesiology.

[29] Defalco J., Steiger D., Gustafson A., Emerling D.E., Kelly M.G., Duncton M.A. (2010). Oxime Derivatives Related to AP18: Agonists and Antagonists of the TRPA1 Receptor. Bioorg. Med. Chem. Lett..

[30] Chen J., Joshi S.K., Didomenico S., Perner R.J., Mikusa J.P., Gauvin D.M., Segreti J.A., Han P., Zhang X.F., Niforatos W. (2011). Selective Blockade of TRPA1 Channel Attenuates Pathological Pain without Altering Noxious Cold Sensation or Body Temperature Regulation. Pain.

[31] Skerratt S. (2017). Recent Progress in the Discovery and Development of TRPA1 Modulators. Prog. Med. Chem..

[32] Logashina Y.A., Solstad R.G., Mineev K.S., Korolkova Y.V., Mosharova I.V., Dyachenko I.A., Palikov V.A., Palikova Y.A., Murashev A.N., Arseniev A.S. (2017). New Disulfide-Stabilized Fold Provides Sea Anemone Peptide to Exhibit Both Antimicrobial and TRPA1 Potentiating Properties. Toxins.

[33] Mitchell M.L., Shafee T., Papenfuss A.T., Norton R.S. (2019). Evolution of Cnidarian Trans-Defensins: Sequence, Structure and Exploration of Chemical Space. Proteins.

[34] Calabrese E.J., Blain R.B. (2011). The Hormesis Database: The Occurrence of Hormetic Dose Responses in the Toxicological Literature. Regul. Toxicol. Pharmacol..

[35] Cookman C.J., Belcher S.M. (2014). Classical Nuclear Hormone Receptor Activity as a Mediator of Complex Concentration Response Relationships for Endocrine Active Compounds. Curr. Opin. Pharmacol..

[36] Andreev Y.A., Osmakov D.I., Koshelev S.G., Maleeva E.E., Logashina Y.A., Palikov V.A., Palikova Y.A., Dyachenko I.A., Kozlov S.A. (2018). Analgesic Activity of Acid-Sensing Ion Channel 3 (ASIC3) Inhibitors: Sea Anemones Peptides Ugr9-1 and APETx2 versus Low Molecular Weight Compounds. Mar. Drugs.

[37] Andreev Y.A., Kozlov S.A., Vassilevski A.A., Grishin E. (2010). V Cyanogen Bromide Cleavage of Proteins in Salt and Buffer Solutions. Anal. Biochem..

[38] Mirdita M., Schütze K., Moriwaki Y., Heo L., Ovchinnikov S., Steinegger M. (2022). ColabFold: Making protein folding accessible to all. Nat. Methods.

