# Supplementary material for: Analysis of Structural Determinants of Peptide MS 9a-1 Essential for Potentiating of TRPA1 Channel
Source: Mar Drugs. 2022 Jul 21;20(7):465. doi: 10.3390/md20070465 (PMC9320628; doi:10.3390/md20070465)
Supplement: Supplementary file 1 [file marinedrugs-20-00465-s001.zip › marinedrugs-1819428-supplementary.pdf]

Supplementary Materials

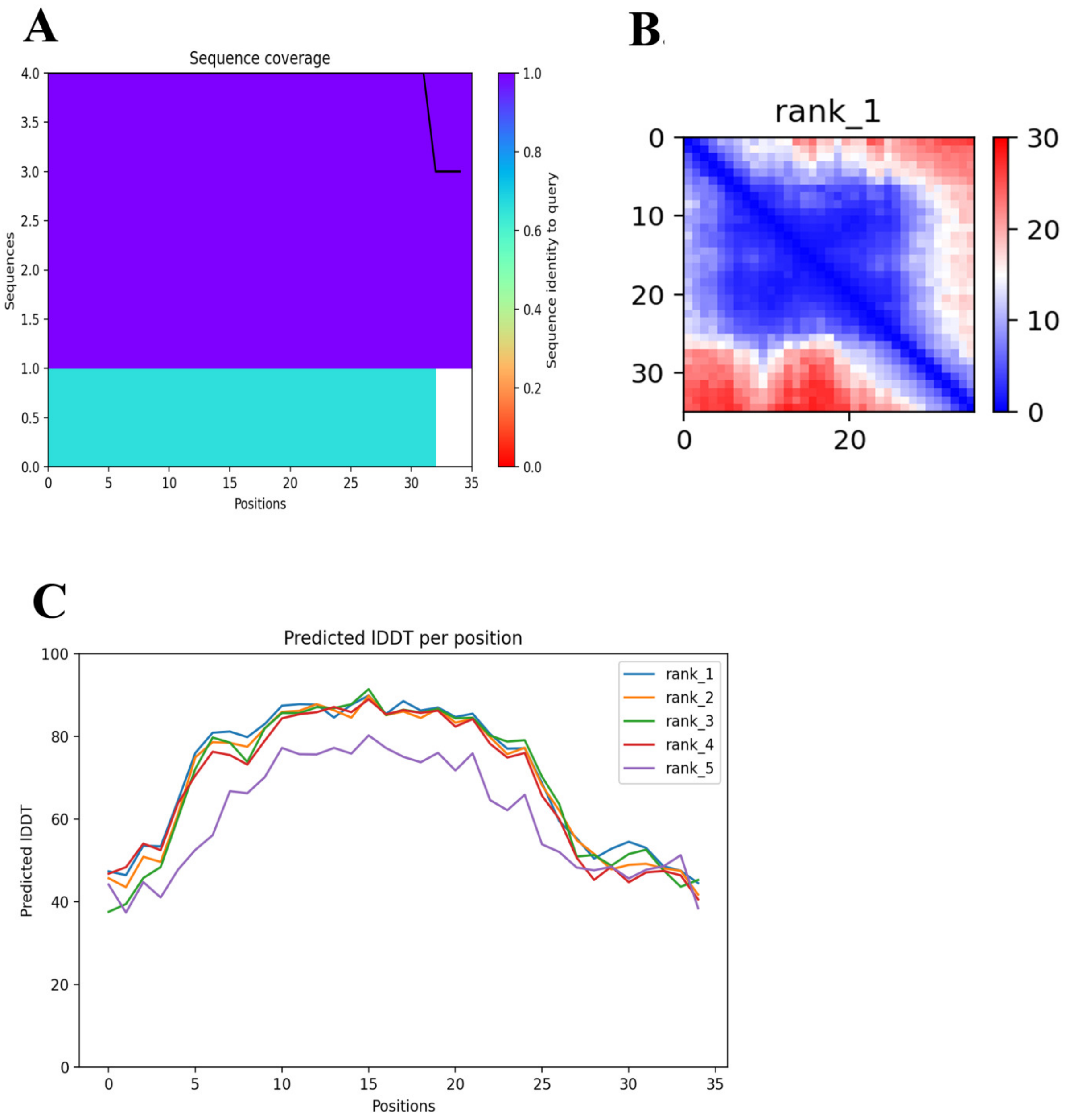

Figure S1. AlphaFold2 Colab quality plots for spatial structure model of Ms9a-1. (**A**). Number of sequences per position. (**B**). Predicted Alignment Error - AlphaFold's expected position error at residue x, when the predicted and true structures are aligned on residue y. The lower is the better. C. Predicted lDDT (Local Distance Difference Test) per position - model confidence (out of 100) at each position. The higher is the better.

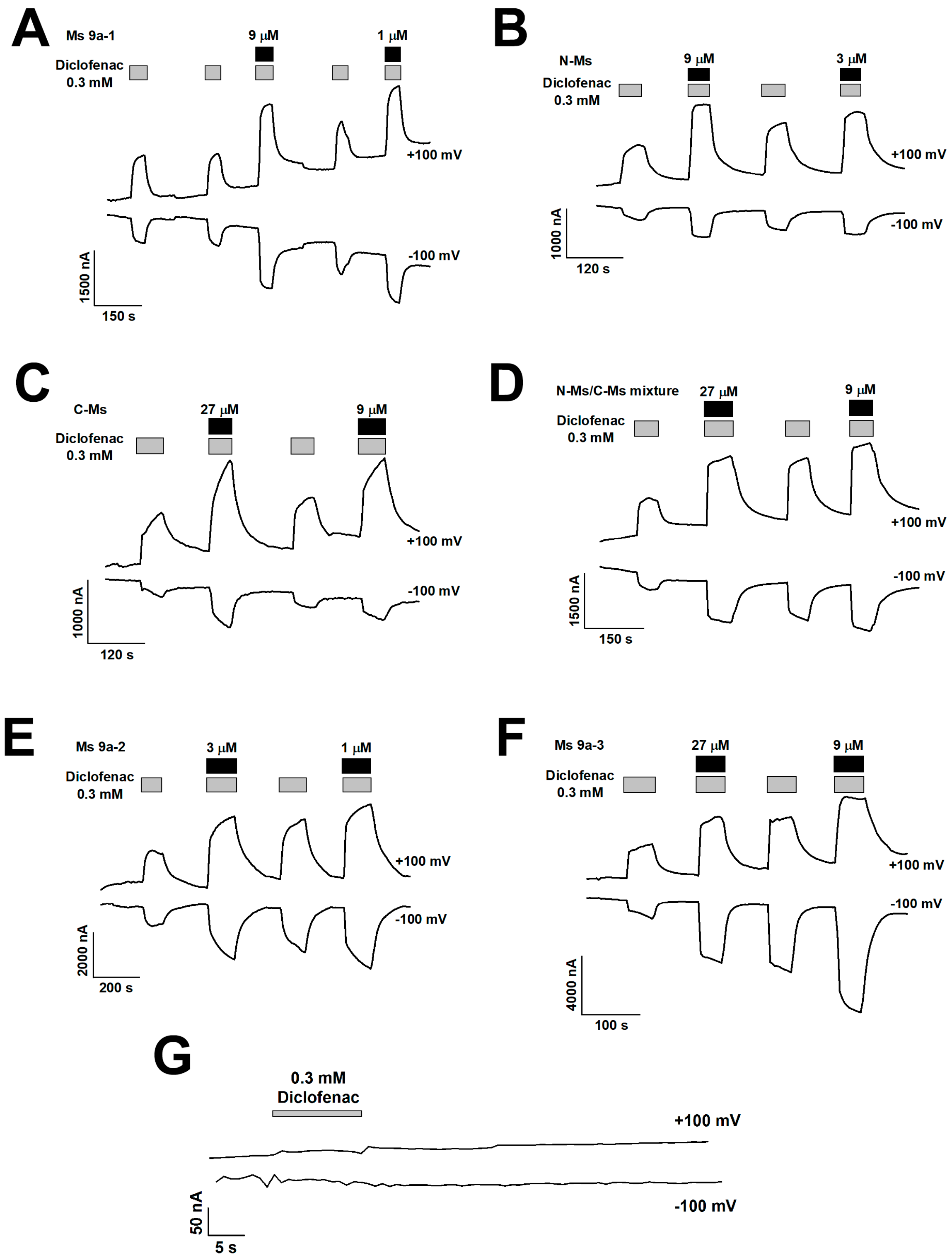

Figure S2. Action of peptides on diclofenac-induced inward and outward TRPA1 currents. Representative traces of Ms 9a-1 **(A)**, N-Ms **(B)**, C-Ms **(C)**, N-Ms+C-Ms **(D)**, Ms9a-2 **(E)**, and Ms9a-3 **(F)** effects on whole-cell currents through *X. laevis* oocytes expressing rTRPA1. **G**, representative trace of diclofenac action on uninjected oocytes of *X. laevis*. The currents were evoked by 0.3 mM diclofenac and were recorded at +100 mV and −100 mV.
